# Neuromuscular electrical stimulation on hearing loss caused by skull base fracture: A protocol for systematic review of randomized controlled trial

**DOI:** 10.1097/MD.0000000000014650

**Published:** 2019-02-22

**Authors:** Lin-hong Yang, Wei-feng Wang, Shu-hong Zhang, Zong-xian Fan, Jian-qi Xiao

**Affiliations:** a Department of Otorhinolaryngology; b Department of Neurosurgery, First Affiliated Hospital of Jiamusi University; c Department of Biology, Basic Medicine School of Jiamusi University, Jiamusi; d Department of Neurosurgery, The First Hospital of Qiqihar City, Qiqihar, China.

**Keywords:** effectiveness, hearing loss, neuromuscular electrical stimulation, randomized controlled trial, safety, skull base fracture, systematic review

## Abstract

**Background:**

This systematic review aims to investigate the effectiveness and safety of neuromuscular electrical stimulation (NMES) on hearing loss (HL) caused by skull base fracture (SBF).

**Methods:**

We will retrieve the following electronic databases of Cochrane Library, PUBMED, EMBASE, Cumulative Index to Nursing and Allied Health Literature, Allied and Complementary Medicine Database, and Chinese Biomedical Literature Database from the inception to January 1, 2019 for relevant RCTs of NMES for HL caused by SBF. Two experienced authors will independently perform the study selection, data extraction, and methodology quality assessment. A 3rd author will solve any disagreements between 2 authors through discussion.

**Results:**

This study will provide a high-quality synthesis of latest evidence of NMES for HL caused by SBF from comprehensive assessments, including hearing loss evaluation, hearing threshold, quality of life, and any relevant adverse events.

**Conclusion:**

The expected results of this systematic review will provide the up-to-date evidence to assess the effectiveness and safety of NEMS for patients with HL caused by SBF.

**Ethics and dissemination:**

The results of this study will be disseminated through publication in a peer-reviewed journal or will be presented at an associated conference meeting. This study will not use individual patient data, thus, the ethical approval is not needed.

**PROSPERO registration number:**

PROSPERO CRD42019120195.

## 1 Introduction

Traumatic brain injury is a leading cause of death and disability.^[1–3]^ Skull fractures are relatively common following head trauma.^[4–6]^ Of these, 4–20% of such fractures are occurred at the base of skull.^[7,8]^ Many reasons that can result in skull base fracture (SBF), such as motor vehicle accidents, fall from heights, and blunt trauma.^[9–12]^ It mainly manifests with hearing loss (HL), cranial nerve deficit, headache, behavioral changes, and hemiparesis, as well as others,^[13–15]^ especially for the HL. If it is not treated fairly and effectively, it can greatly affect the quality of life in patients who experience such condition.^[16,17]^

A variety of clinical studies have reported that neuromuscular electrical stimulation (NMES) can be utilized to treat HL, and have achieved very promising outcomes.^[18–24]^ However, up to date, no study has systematically assessed the effectiveness and safety of NMES for the treatment of HL caused by SBF. Therefore, in this systematic review, we will firstly evaluate the effectiveness and safety of NMES for patients with HL caused by SBF.

## 2 Methods

### 2.1 Inclusion criteria for study selection

#### 2.1.1 Type of studies

All relevant randomized controlled trials (RCTs) regarding NMES for the treatment of HL caused by SBF will be included without any language and publication status restrictions. However, non-clinical trials, non-control studies, non-RCTs and Quasi-RCTs will not be included.

#### 2.1.2 Type of participants

All participants who are clinically diagnosed with HL caused by SBF will be included regardless the age, sex, and race. However, they will be excluded if they had HL before the SBF, or result from other disorders or diseases, except the SBF.

#### 2.1.3 Type of interventions

The intervention of experimental group should utilize NMES only. The combination of NMES with other therapies is not allowed in this systematic review. The interventions in the control group can be any kinds of therapies, but not any forms of NMES.

#### 2.1.4 Type of outcomes

The primary outcome is hearing loss, as assessed according to the Brock grade, acceptable noise level test, or other related scales. The secondary outcomes are hearing threshold, as measured by pure-tone audiometry, speech audiometry or other tools; and quality of life, as measured by the 36-Item Short Form Health Survey or other scales. Any relevant adverse events are also evaluated.

### 2.2 Search strategy

Cochrane Library, PUBMED, EMBASE, Cumulative Index to Nursing and Allied Health Literature, Allied and Complementary Medicine Database, and Chinese Biomedical Literature Database will be searched from the inception to January 1, 2019 for relevant RCTs of NMES for HL caused by SBF. Detailed strategy for searching the Cochrane Library database is presented in Table 1. Similar strategies will be applied to the other databases.

**Table 1:**
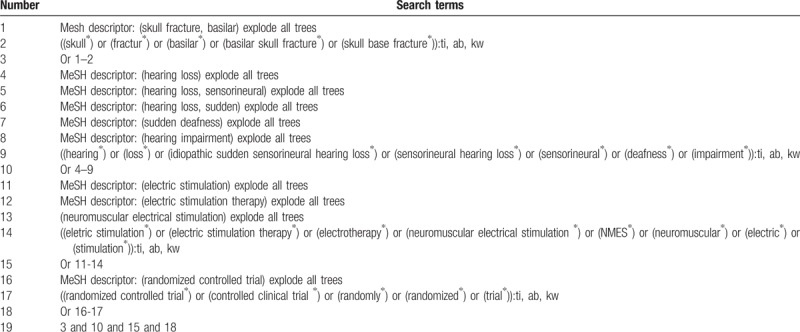
Search strategy applied in Cochrane Library database.

Additionally, we will also manually search the references lists of relevant trials and reviews for any other potential eligible literatures. Besides, the websites of clinical trials registry will also be considered to search.

### 2.3 Data collection and analysis

#### 2.3.1 Study selection

**Figure 1:**
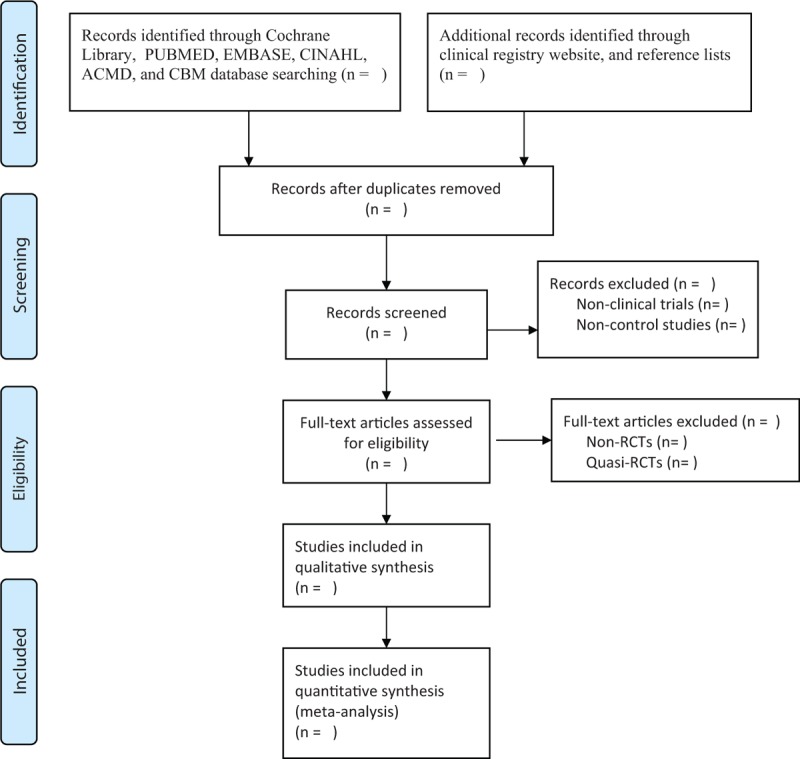
Flowchart of study selection process.

Two experienced authors will independently search all databases and select studies by scanning titles, abstracts, and full texts reading based on the previous eligibility criteria. Studies will be excluded with specific exclusion reasons. Any disagreements will be solved by group discussion with other authors. The whole selection process will follow the Preferred Reporting Items for Systematic Review and Meta-analysis flow diagram, and will be presented in Figure 1.

#### 2.3.2 Data extraction and missing data management

After study selection, the same 2 authors will independently perform the data extraction by using previous designed data extraction forms. Any divided options regarding the data extraction will be resolved through discussion with a 3rd experienced author. If some important information is missing or not reported in the primary study, the original authors of the primary studies will be contacted to request this information, including the missing data. If such information can not be achieved, then we will analyze the available data.

#### 2.3.3 Risk of bias assessment

Cochrane risk of bias tool will be adopted to assess the risk of bias for each included study. Each included study will be assessed at 7 domains, and each domain will be classified as high, unclear or low risk of bias. Two experienced authors will be independently in charge of the assessment. We will reach a consensus through discussion or consult with a 3rd experienced author involved if there are disagreements between 2 authors.

### 2.4 Data synthesis and analysis

#### 2.4.1 Measurement of treatment effect

For continuous outcomes, the extracted data will be presented as mean difference with 95% confidence intervals (CIs). As for dichotomous outcomes, the extracted data will be computed as risk ratio with 95% CIs.

#### 2.4.2 Assessment of heterogeneity

Heterogeneity is identified by *I*^*2*^ index and Cochrane Q statistic test. It among included studies will be detected to assess the feasibility to pool the data, and to conduct meta-analysis. If the value of *I*^*2*^ is over 50%, the substantial heterogeneity will be considered, and subgroup analysis will be performed to investigate the potential factors from clinical or methodological heterogeneity.

#### 2.4.3 Data synthesis

RevMan 5.3 Software will be used to pool the data and conduct the meta-analysis. If the value of *I*^*2*^ is less than 50%, the fixed-effect model will be used for data synthesis, and meta-analysis will be conducted. If not, random-effect model will be employed for data synthesis. Meanwhile, subgroup analysis and sensitivity analysis will also be carried out. If it is still not available to pool the data and to perform a meta-analysis after the subgroup analysis, we will only elaborate the summary description instead.

#### 2.4.4 Subgroup analysis

Subgroup analysis will be carried out to explore the sources of heterogeneity according to the study locations, patient characteristics, treatment dosage or duration, types of treatments, or controls, as well as different outcome measurement scales.

#### 2.4.5 Sensitivity analysis

If the data is available to be pooled, sensitivity analysis will be adopted to assess the robustness of pooled results data, missing data, and methodological quality.

#### 2.4.6 Assessment of reporting bias

If the number of included trials is sufficient (over 10 studies) in this review, the funnel plot and Egg's regression test will be carried out to detect the possible reporting bias.

## 3 Discussion

The HL, with a large population affected, is a global epidemic issue during the past decades. Lots of researches have reported that NMES is effective for the HL treatment. However, no systematic review has addressed to investigate the effectiveness and safety of NMES on HL caused by SBF. Therefore, it is very necessary to conduct the present systematic review. It will be performed according to the Cochrane Handbook to ensure that it can provide helpful information and evidence for both clinicians and patients with HL.

## Author contributions

**Conceptualization:** Lin-hong Yang, Wei-feng Wang, Zong-xian Fan, Jian-qi Xiao.

**Data curation:** Lin-hong Yang, Wei-feng Wang, Shu-hong Zhang, Zong-xian Fan, Jian-qi Xiao.

**Formal analysis:** Lin-hong Yang, Wei-feng Wang, Zong-xian Fan.

**Funding acquisition:** Jian-qi Xiao.

**Investigation:** Jian-qi Xiao.

**Methodology:** Lin-hong Yang, Wei-feng Wang, Shu-hong Zhang, Zong-xian Fan.

**Project administration:** Jian-qi Xiao.

**Resources:** Lin-hong Yang, Wei-feng Wang, Shu-hong Zhang, Zong-xian Fan.

**Software:** Lin-hong Yang, Wei-feng Wang, Zong-xian Fan.

**Supervision:** Jian-qi Xiao.

**Validation:** Lin-hong Yang, Wei-feng Wang, Jian-qi Xiao.

**Visualization:** Lin-hong Yang, Shu-hong Zhang, Zong-xian Fan, Jian-qi Xiao.

**Writing – original draft:** Lin-hong Yang, Wei-feng Wang, Shu-hong Zhang, Jian-qi Xiao.

**Writing – review & editing:** Lin-hong Yang, Shu-hong Zhang, Zong-xian Fan, Jian-qi Xiao.
